# Likely Geographic Distributional Shifts among Medically Important Tick Species and Tick-Associated Diseases under Climate Change in North America: A Review

**DOI:** 10.3390/insects12030225

**Published:** 2021-03-05

**Authors:** Abdelghafar Alkishe, Ram K. Raghavan, Andrew T. Peterson

**Affiliations:** 1 Biodiversity Institute, University of Kansas, Lawrence, KS 66045, USA; 2 Zoology Department, Faculty of Science, University of Tripoli, Tripoli, Libya; 3 Center for Vector-borne and Emerging Infectious Diseases, Departments of Veterinary Pathobiology and Public Health, College of Veterinary Medicine and School of Health Professions, University of Missouri, Columbia, MO 65211, USA; raghavanrk@missouri.edu

**Keywords:** potential geographic distribution, ecological niche modeling, current and future scenarios, Mexico, United States, Canada

## Abstract

**Simple Summary:**

North America is considered as an area likely to be significantly affected by global warming, with climate change causing markedly warmer winter temperatures in the United States in recent decades. Ticks are sensitive to changes in ambient abiotic conditions and, therefore, climate: they are poikilothermic, with the life stages of each species requiring specific sets of environmental conditions for successful development and survival. Our review focuses on (1) identifying and exploring suitable areas for the eight medically important vector tick species in North America; (2) exploring whether and how species’ distributions are likely to shift in coming decades in response to climate change, and in what ways; (3) and providing a picture on the status of the tick-associated diseases in North America from the present to the future.

**Abstract:**

Ticks rank high among arthropod vectors in terms of numbers of infectious agents that they transmit to humans, including Lyme disease, Rocky Mountain spotted fever, Colorado tick fever, human monocytic ehrlichiosis, tularemia, and human granulocytic anaplasmosis. Increasing temperature is suspected to affect tick biting rates and pathogen developmental rates, thereby potentially increasing risk for disease incidence. Tick distributions respond to climate change, but how their geographic ranges will shift in future decades and how those shifts may translate into changes in disease incidence remain unclear. In this study, we have assembled correlative ecological niche models for eight tick species of medical or veterinary importance in North America (*Ixodes scapularis*, *I. pacificus*, *I. cookei*, *Dermacentor variabilis*, *D. andersoni*, *Amblyomma americanum*, *A. maculatum*, and *Rhipicephalus sanguineus*), assessing the distributional potential of each under both present and future climatic conditions. Our goal was to assess whether and how species’ distributions will likely shift in coming decades in response to climate change. We interpret these patterns in terms of likely implications for tick-associated diseases in North America.

## 1. Introduction

**Figure 1:**
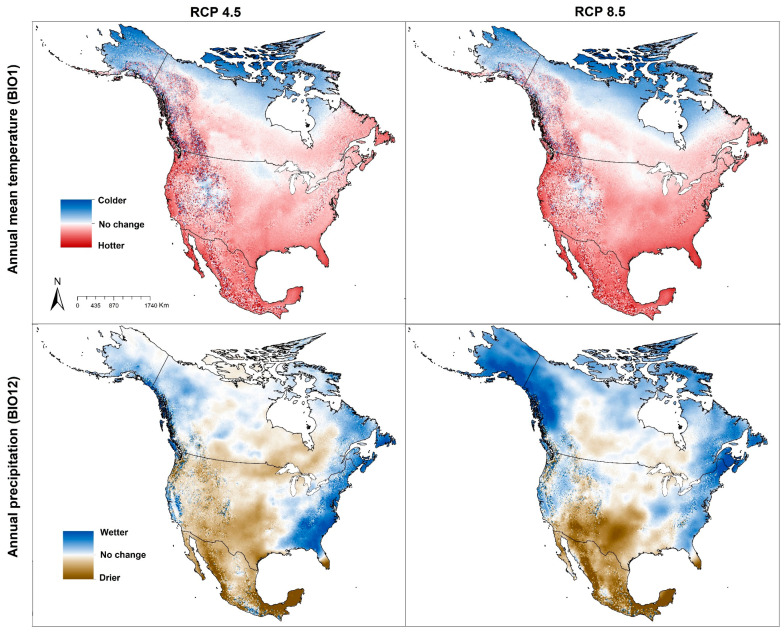
Climate variables for present (white color = no change) and future conditions (National science foundation department of energy, national center for atmospheric research, USA). General circulation model (GCM: CESM1) under representative concentration pathway (RCP 4.5 and RCP 8.5) for the year 2050.

Ticks spend parts of their life cycle on and off of their blood-meal hosts, and are obligate blood feeders [1]. Since ticks spend much of their life cycle exposed to the environments, they must respond to local conditions including abiotic (e.g., humidity, temperature, soil moisture) and biotic factors (e.g., humid leaf, dense vegetation, dense shade, host interaction) [2]. Given that the ticks need a blood meal at each stage (larva, nymph, adult), the effects of abiotic factors on tick populations and distributions are often more immediate than biotic factors [3]. North America is considered as an area likely to be affected significantly by global warming, with climate change causing markedly warmer winter temperatures in the United States [4], Canada [5], and Mexico [6] in recent decades. The increasing temperature trends as a result of climate change will probably lead to the expansion of and/or shifts in the potential distributions of several tick species, generally towards higher latitudes and higher elevations (Figure 1) [7].

The geographic ranges of 25 tick species of medical and veterinary importance have been described for regions globally [2]. Ten species of hard and soft ticks are known to bite and transmit disease to humans in the United States [7]. The U.S. Centers for Disease Control and Prevention (CDC) has developed risk maps for different tick species based on a regional scale [8]; however, only a few, scattered studies have investigated distributional changes that would be expected under climate change of these species [9,10,11,12,13,14]. 

In recent years, several new tick-associated pathogens have been documented, and tick vectors and tick-associated diseases have expanded geographically into new areas around the world [15]. In the United States, cases of tick-associated diseases (including Lyme disease, spotted fever group rickettsiosis or SFGR, babesiosis, Powassan virus, anaplasmosis/ehrlichiosis, and tularemia) totaled 48,610 cases in 2016 and 59,349 in 2017, which were the highest numbers of cases recorded since recording began [16]. Seven new pathogens (*Borrelia mayonii*, *B. miyamotoi*, *Ehrlichia ewingii*, *E. muris eauclairensis*, Heartland virus, *Rickettsia parkeri*, and *Rickettsia* species 364D) have been discovered in the United States in the last two decades by using advanced molecular detection (AMD) program that detects DNA of new pathogens in humans and animals [16]. In Canada, *B. miyamotoi* and *B. mayonii* were identified as new disease agents in 2013 and 2014, respectively [17]. In Mexico, documentation of cases of the most common tick-associated disease, Rocky Mountain spotted fever (RMSF), has expanded from northern Mexico into 30 states across the country [18].

The purpose of this paper is to explore and highlight potential distributional changes of tick species of medical or veterinary importance under climate change in North America (Figure 1). Our goal is to illuminate possible cross-species trends in the distribution and status of tick-associated diseases across the region, particularly as regards the hypotheses that climate warming will shift species’ distributions to higher latitudes and higher elevations. We selected tick species that are known to bite humans and transmit diseases to people based on information from the U.S. Centers for Disease Control and Prevention (CDC) [19]. Tick species analyzed herein include Western blacklegged tick (*Ixodes pacificus*), Blacklegged tick (*I. scapularis*), Groundhog tick (*I. cookei*), Rocky Mountain wood tick (*Dermacentor andersoni*), American dog tick (*D. variabilis*), Gulf coast tick (*Amblyomma maculatum*), Lone star tick (*A. americanum*), and Brown dog tick (*Rhipicephalus sanguineus*). 

## 2. Tick-Associated Diseases of Humans in North America

### 2.1. Lyme Disease

*Borrelia burgdorferi sensu stricto* is the causative agent of Lyme disease, transmitted by *Ixodes pacificus* and *I. scapularis* [20]. Lyme disease was recognized as an important infectious disease in the late 20th century; since that time, CDC reported an increase in Lyme disease cases in several parts of the United States. For example, in 2000, 18,000 cases of Lyme disease were documented [21]; however, numbers of cases of the disease reached 42,743 in 2017 and 33,666 in 2018 [22]. In the United States, risk of Lyme disease increased 300% in the northeastern and midwestern parts of the country [23]. Canada has also seen increased Lyme case rates, rising from 144 in 2009 to 917 in 2015, respectively, all reported from eastward of Manitoba [24]. In Mexico, although Lyme disease is not well studied in terms of its biogeography and epidemiology [25], human cases have been documented in the northeastern part of the country and in Mexico City [26]. 

### 2.2. Babesiosis

The protozoan genus *Babesia* (e.g., species *B. microti*, *B. duncani*, *B. divergens*, and *B. venatorum*) is the causative agents of babesiosis, which can cause influenza-like symptoms (chills, fever, headache, fatigue, and body aches) and thrombocytopenia [27]. It is transmitted by bites of the ixodid tick *Ixodes scapularis* [28,29,30]. In the United States, this disease is found in the Northeast and Midwest, with a total of 7612 cases documented in the period between 2011 and 2015; highest incidence rates of babesiosis (7194 of the total 7612 cases) were in seven states (Massachusetts, Connecticut, New York, Rhode Island, Wisconsin, New Jersey, and Minnesota), whereas Maine and New Hampshire documented <200 cases over 5 years [31]. In 2016, 2017, and 2018, across the United States, CDC documented 1910, 2368, and 2160 new cases, respectively [22]. In Canada, babesiosis is not a nationally notifiable disease; however, 1119 cases were reported between 2011 and 2017 in many provinces (Alberta, British Columbia, Manitoba, New Brunswick, Nova Scotia, Newfoundland and Labrador, Ontario, Prince Edward Island, Québec, Saskatchewan) [32]. In Mexico, babesiosis is poorly studied; however, since the first reported human case in 1976, only four cases were reported in 2015 in Yucatán state [33].

### 2.3. Anaplasmosis and Ehrlichiosis

*Anaplasma phagocytophilum* and *Ehrlichia* species (e.g., *E. chaffeensis*) are bacterial pathogens that threaten human health [34]. Anaplasmosis and ehrlichiosis are the second most frequently recorded tick-associated diseases (after Lyme disease) in the United States [22], but carry higher fatality rates (2–5%) than Lyme disease [17,35]. For ehrlichiosis, numbers of documented cases rose from 338 in 2004 to 1377 in 2006, with 7309 cases between 2013 and 2017, most from four states (New York, Virginia, Missouri, and Arkansas) [36]. Anaplasmosis cases also rose from 537 in 2004 to 4151 cases in 2016 [36], most from eight states (Vermont, Maine, Rhode Island, Minnesota, Massachusetts, Wisconsin, New Hampshire, and New York) [37]. Numbers of anaplasmosis and ehrlichiosis cases have also increased in Canada since 2013, although relatively few cases have been recognized compared to numbers in the United States [38]. 

### 2.4. Spotted Fever Rickettsiosis (SFR)

Some members of the bacterial genus *Rickettsia* cause disease in humans, including *R. rickettsii* which causes Rocky Mountain spotted fever (RMSF) and is transmitted by several tick species, such as *Dermacentor variabilis*, *D. andersoni*, and *Rhipicephalus sanguineus* [39]; *Rickettsia parkeri*, which causes disease similar to RMSF but milder, is transmitted by *Amblyomma maculatum* [40]. In 2010, CDC included all rickettisial diseases (RMSF, *Rickettsia parkeri* rickettsiosis, Pacific Coast tick fever, and rickettsialpox) in a single category called spotted fever rickettsiosis (SFR) [41]. The states most affected by SFR, with 50% of all cases, are Oklahoma, Missouri, Arkansas, North Carolina, Virginia, and Tennessee; Arizona became a new state under consideration with >360 cases and 21 deaths between 2003 and 2016 [41]. Between 1997 and 2002, 3600 RMSF cases were documented in the United States [42]. CDC reported 6248 and 5544 spotted fever rickettsiosis cases in 2017 and 2018, respectively. In Canada, although *R. rickettsii* was isolated from *D. andersoni* in Alberta and British Columbia in 1942 [43], SFR is less well documented compared to in the United States, perhaps as a function of lower vector species diversity [44]. Several studies have reported that *R. rickettsii* transmission is low given the low frequency of detection of the bacterial agent in tick vectors [45,46]. In Mexico, RMSF was identified after outbreaks in Sonora, Sinaloa, Coahuila, and Durango in the 1940s, with fatality rates of 30–80% of reported cases [47]. Those outbreaks continued to happen across northern Mexico thanks to the presence of the host tick *Rhipicephalus sanguineus* sensu lato; in 2003–2016 and 2009–2016, the states of Sonora and Baja California registered 1394 and 967 RMSF cases, and 247 and 132 deaths, respectively [47].

### 2.5. Tularemia

Tularemia is a disease caused by the bacterium *Francisella tularensis*, which can cause health problems related to the skin, eyes, lungs, and lymph nodes [48]. Five subspecies of *F. tularensis* have been described, but only subspecies *F. t. tularensis* and *F. t. holarctica* cause human disease in North America [49]. This disease can be transmitted by bites of insects such as ticks (*Dermacentor variabilis*, *D. andersoni*, and *Amblyomma americanum*), deer flies (*Chrysops* spp.), physical contact with infected animals, or drinking contaminated water [50]; in the central United States, biting flies are relatively rare, so most risk is related to tick bites (particularly *D. andersoni*) and animal contact [51]. In the 1930s and 1940s, tularemia cases numbered >1000 cases per year in the United States [52]. Problems emerged when tularemia was removed from the list of nationally reportable diseases (it came back on the list in 2000 out of concern about the possibility of bioterrorism) [53]. In recent years, however, numbers of tularemia cases in the United States reached >200 cases/year [22]; tularemia is rare in Canada, with 6–22 cases documented yearly during 2005–2011 [54]. Tularemia cases were originally concentrated in the south-central United States, yet since 1965, it expanded to more northern states [55]. In the United States, through 2001–2010 and 2010 surveys, 59% and 65% (respectively) of total tularemia cases were documented in six states (Arkansas, Kansas, Missouri, Oklahoma, Massachusetts, and South Dakota) [51]; by 2015, four states (Wyoming, Colorado, Nebraska, and South Dakota) documented increasing numbers of cases [56]. A detailed analysis of the geography/environment of these case distributions indicated that the range-shift trends were consistent with expectations deriving from observed climate trends across the United States [57].

### 2.6. Powassan Virus

Powassan virus (genus *Flavivirus*) group is one of the tick-borne encephalitis viruses, and causes severe neurological damage with high case fatality rates [58]. In 1958, the first human case was documented from Powassan, Ontario, Canada [59], with the virus isolated from brain tissue of a 5-year-old boy, after he died from severe encephalitis [60]. The main enzootic cycle for Powassan virus involves *Ixodes cookei* as the vector, and groundhogs (*Marmota monax*) or striped skunks (*Mephitis mephitis*) as reservoir hosts [61]. Although POWV is rare, numbers of cases have increased in recent years; in all, 13 states reported POWV cases during 2010–2019: Connecticut, Indiana, Maine, Massachusetts, Minnesota, New Hampshire, New Jersey, New York, North Carolina, North Dakota, Pennsylvania, Rhode Island, and Wisconsin. Minnesota, Massachusetts, Wisconsin, and New York have had highest numbers of POWV cases in recent years [62]. In Canada, 21 cases have been reported since 1958, and most recorded POWV cases were from the Great Lakes Region, and fewer cases were from the Maritime Provinces [63]. No cases have been documented from Mexico.

## 3. Methods

This contribution aims to provide an overview of present and likely future geographic distributions of medically important tick vector species. To that end, we take advantage of several papers that are already published by our research group [12,13,14], and add several additional analyses for other tick species that are at various stages of preparation for publication. The advantage of using a suite of studies that comes from a single research group is that the methods are mostly coincident, although a few improvements and modifications have certainly been added along the way in the process of assembling this body of work [64]. As such, we offer here a broad outline of the methods that we used and refer the reader to the original publications [12,14] and to the Supplementary Information for more details.

We chose tick species for analysis based on their known or suspected roles as vectors in transmitting tick-borne pathogens to humans (Table 1) [19]. We obtained occurrence data for each tick species of interest from online sources including the Global Biodiversity Information Facility (http://www.gbif.org, accessed on 20 January 2020), VectorMap (http://vectormap.si.edu/, accessed on 20 January 2020), and BISON (https://bison.usgs.gov, accessed on 20 January 2020); we also surveyed the relevant scientific literature for additional occurrence data [65,66,67,68,69,70,71,72,73,74]. To relate known occurrences of each tick species to current environmental conditions, we obtained climate average data from WorldClim (http://www.worldclim.org, accessed on 20 January 2020). We characterized scenarios of future climate conditions via multiple general circulation models (GCMs; data layers from Climate Change, Agriculture and Food Security, CCAFS; http://www.ccafs-climate.org/data_spatial_downscaling, accessed on 20 January 2020) and two representative concentration pathways (RCP 4.5, which is a more optimistic view of climate change future, and RCP 8.5, which is less optimistic, and anticipates more severe climate change effects).

We created species-specific model calibration areas that are based on detailed assumptions about the set of areas to which each species has had access over relevant time periods; this set of areas is termed M in the biotic-abiotic-mobility (BAM) framework for distributional ecology [75]. Ecological niche modeling was done in R version 3.5.1 [76] using Maxent 3.4.1 [77], via the kuenm package [64] (available at https://github.com/marlonecobos/kuenm, accessed on 20 January 2020). Models were evaluated in terms of statistical significance compared to null expectations [78], performance in terms of omission rate, and low model complexity [79]. For full details on the methods used, see published studies [12,14] and the Supplementary Materials (File S1).

To provide an empirical basis for assessing model predictions for tick vectors, as they relate to transmission of pathogens to humans, we sought disease case-occurrence data for the regions of interest. We were successful in obtaining detailed data (i.e., county-level totals of case numbers) for five tick-associated diseases for 2017—these data were kindly provided by Kristen Nichols Heitman, and Amy Schwartz (Centers for Disease Control and Prevention, pers. comm.). We visualized predictions and data on maps using ArcMap version 10.5.

## 4. Results

### 4.1. Geographic Distribution of Tick Vector Species

#### 4.1.1. *Ixodes scapularis* (Black-Legged Tick)

Historically (i.e., in the late 1800s and early 1900s), the geographic distribution of *I. scapularis* was restricted in the eastern United States in the face of widespread deforestation and reduced deer populations [94]. In the 20th century, however, with reforestation efforts and increasing deer populations, this species spread broadly to occupy much of the eastern United States and southern Canada [94,95]. Eisen et al. [72] depicted *I. scapularis* as established in 842 counties in 35 states [72]; a new report from CDC showed new counties in the northern United States (especially in North Dakota and Minnesota) as established to increase the number from 842 to 975 counties between 2016 and 2019 [73].

**Figure 2:**
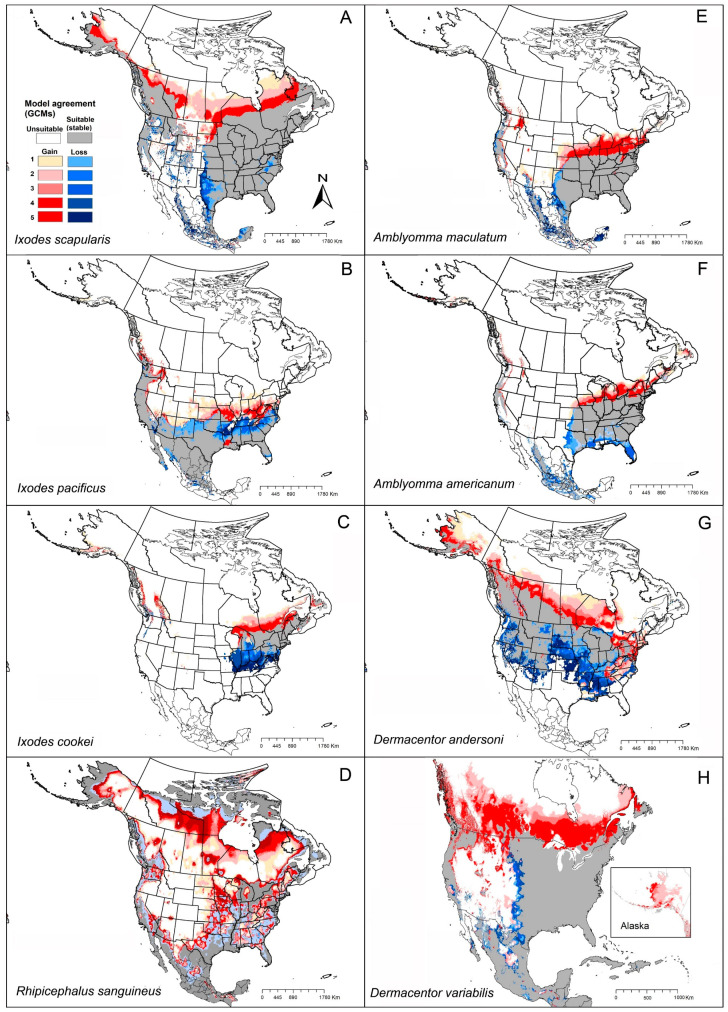
Summary of the potential geographic distributions of eight medically important ticks, both at present and into the future (under RCP4.5). Gray represents stable suitable areas. Red indicates expansion suitable areas under future conditions (dark red = high model agreement, light red = low model agreement). Blue indicates suitable in current time, but not suitable in future (dark blue = high model agreement, light blue = low model agreement).

Our model results showed a broad potential geographic distribution, including the documented distribution across the eastern United States, and some areas in western United States and Canada not known to hold populations in those areas and that are likely outside of the species’ dispersal reach (Figure 2A). In our modeling results, areas of potential range expansion were in areas of northern Minnesota, North Dakota, and South Dakota, as well as areas of northern and western Canada (Figure 2A). Range reductions were anticipated along the southwestern parts of the species’ range, in Texas, as well as in restricted areas of western Kansas, Oklahoma, and Mexico (Figure 2A).

**Figure 3:**
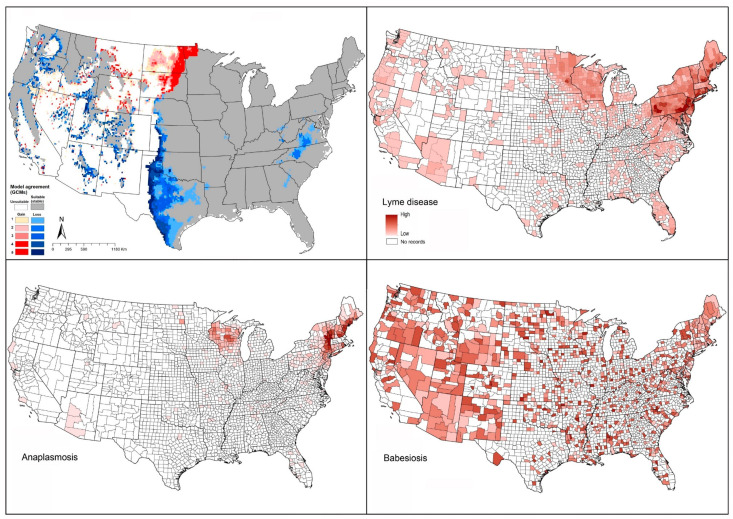
Geographic distribution of *Ixodes scapularis* and diseases that it is suspected to transmit in the United States. Dark pink represents high incidence, light pink indicates low incidence. White represents no records.

We compared case distributions of tick-borne pathogens known to be associated at least in part with this species (Lyme disease, anaplasmosis, and babesiosis) with the modeled suitable areas of *I. scapularis*. For Lyme disease cases, our predictions coincided closely with documented cases: highest numbers of cases were in eastern states (Pennsylvania, New Jersey, Connecticut, Delaware, New Hampshire) and the Midwest (Wisconsin and Minnesota; Figure 3). Anaplasmosis cases show a similar pattern, with highest incidence rates in New England and Wisconsin (Figure 3). Babesiosis cases showed a scattered pattern of occurrence across the United States, coinciding with the potential geographic distribution of *I. scapularis* in the eastern and midwestern states (Figure 3).

#### 4.1.2. *Ixodes pacificus* (Western Blacklegged Tick)

*Ixodes pacificus* is a known vector of Lyme disease, anaplasmosis, and tick-borne relapsing fever in the western United States [96]. This species can be found along the Pacific Coast, in Washington, Oregon, and California, as well as in parts of Arizona, Nevada, and Utah. The species has established populations in 106 (3.6%) of 3141 continental United States counties [72]. The latest update on populations of this species indicated that this number has decreased to 95 counties [73].

Our models showed suitable areas around the states mentioned above, and broadly in the southern states, from New Mexico to Florida, but without any documented presence of this species in those areas (Figure 2B). In Canada, only restricted suitable areas were indicated, in southern British Columbia (Figure 2B). This species seems to have little potential for expansion, with only minor improvements in suitability in eastern Washington, Oregon, California, and northern Arizona, Washington, Idaho, and into Canada. Only highly restricted areas showed anticipated reductions in suitability in New Mexico, Texas, and southeastern states, which means that this species will see mostly stable environmental conditions in areas where it currently has established populations (Figure 2B).

**Figure 4:**
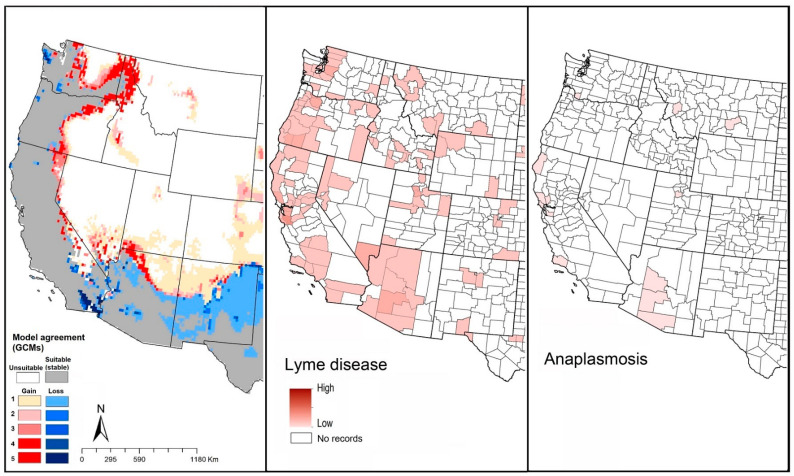
Geographic distribution of *Ixodes pacificus* and the diseases that it likely transmits in the United States. Dark pink represents high incidence, light pink indicates low incidence. White represents no records.

Reported Lyme disease cases showed patterns similar to the model-predicted geographic distribution of *I. pacificus* in California, Oregon, Washington, and Arizona (Figure 4). Anaplasmosis appeared to have less frequent occurrence in the western states compared to eastern states (Figure 3 and Figure 4). Babesiosis showed high concentrations in areas where *I. pacificus* was modeled as seeing suitable conditions (and where it is known to occur) in California, Oregon, Washington, and Arizona (Figure 4). However, we also noted that neighboring states documented babesiosis cases, even though the vector tick species is ostensibly absent.

#### 4.1.3. *Ixodes cookei* (Groundhog Tick or Woodchuck Tick)

*Ixodes cookei* is found broadly in eastern North America, in temperate broadleaf and mixed forests [97]. Based on virus isolations from ticks in eastern North America, this species is known to serve as a significant vector of Powassan encephalitis virus (POWV) [59]. However, *I. scapularis* and *I. marxi* are also considered to be vectors of POWV [62].

Our models showed that the southern range limit of *I. cookei* is in restricted areas of northern Tennessee and North Carolina, and the species is anticipated to occur in southeastern Canada. Missouri and Wisconsin appear to constitute the western limit of the distribution of the species (Figure 2C). Our model predictions identified suitable areas in western Canada and the western United States, where *I. cookei* has been documented as a newly established species in southwestern British Columbia [74]. Our models showed that this species will see a shifting distributional potential extending farther northward into new areas in Michigan and southern Canada (Figure 2C).

**Figure 5:**
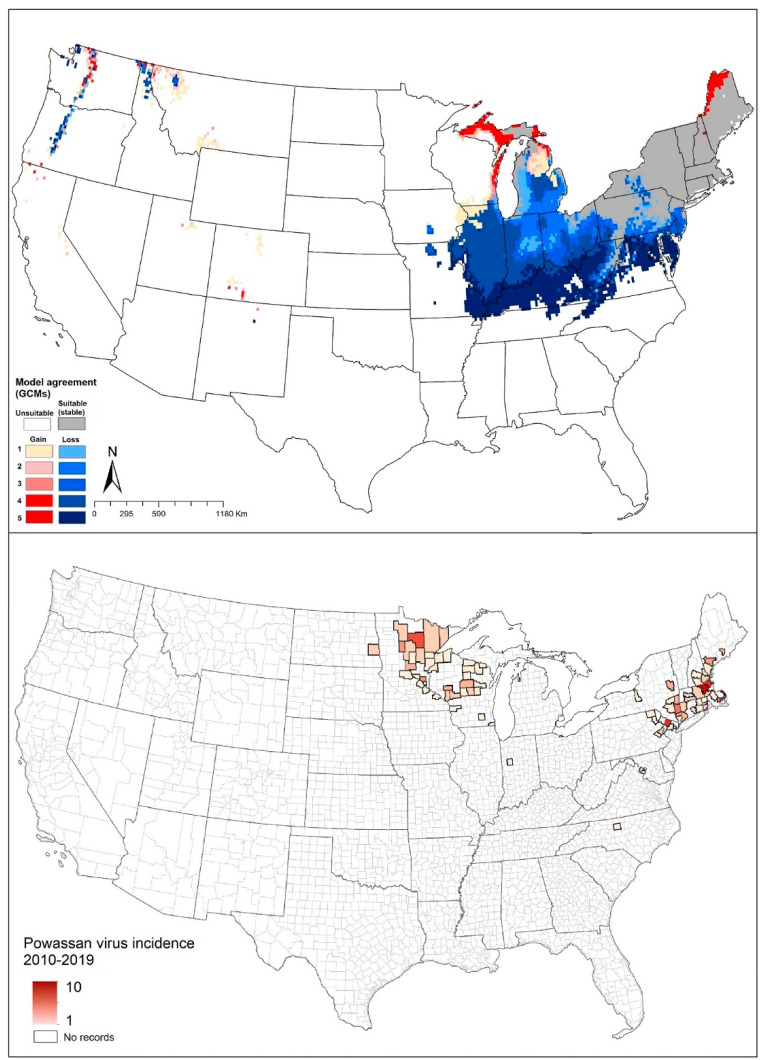
Geographic distribution of *Ixodes cookei* and Powassan encephalitis virus in the United States. Dark pink represents high incidence, light pink indicates low incidence. White represents no records.

Reported cases of Powassan encephalitis virus showed a generally close correspondence with the modeled potential distribution of *I. cookei*, particularly across the northeastern United States (Figure 5). However, the high observed incidence in Minnesota and Wisconsin fell in areas not identified by our models as suitable for *I. cookei* (Figure 5). This latter outcome supports the idea that another tick species (perhaps *I. scapularis*) might be serving as the primary vector of POWV in those areas.

#### 4.1.4. *Rhipicephalus sanguineus* (Brown Dog Tick)

*Rhipicephalus sanguineus* is a vector of many pathogens, including bacteria, viruses, protozoa, and helminths [98]. The best-known pathogens that *R. sanguineus* transmits include *Coxiella burnetii*, *Ehrlichia canis*, *Rickettsia conorii*, and *R. rickettsii* [99]. In North America, this species is also responsible for transmission of RMSF in the southwestern United States and in northern Mexico [8]. This species is among the best-studied species thanks to its zoonotic concern; its uses dogs and other animals including humans as hosts [86].

*Rhipicephalus sanguineus* can be found in many habitats across its broad, near-global distribution [86]. A recent CDC map indicated that this species occurs pretty much ubiquitously across the United States [8]. A recent analysis by Alkishe et al., (2020) found suitable areas for *R. sanguineus* from the southwestern to northeastern United States; most of Mexico is also apparently suitable for this species (Figure 2D). Under future conditions, the modeled potential distribution of this species showed different results between RCP 4.5 and RCP 8.5: the species is anticipated to lose suitable area under RCP 4.5, but to gain suitable area northward in the Midwest and southern United States under RCP 8.5 (Figure 2D and Figure S2).

**Figure 6:**
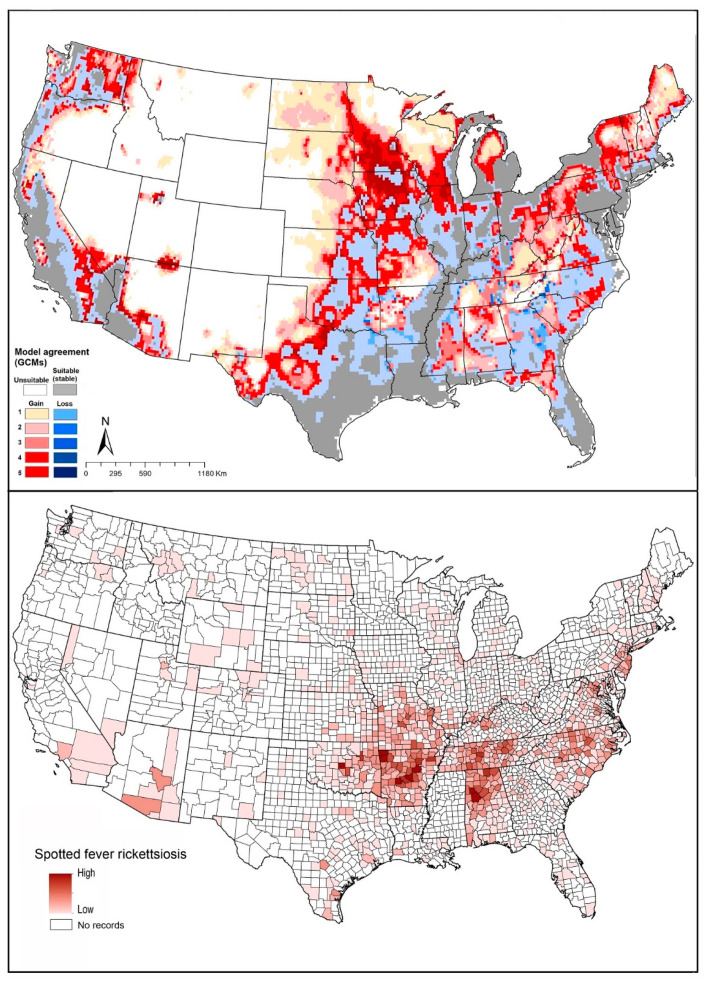
Geographic distribution of *Rhipicephalus sanguineus* and the disease that it likely transmits in the United States. Dark pink represents high incidence, light pink indicates low incidence. White represents no records.

*Rhipicephalus sanguineus* is the prime vector of RMSF in the western states (California, Nevada, Arizona, and New Mexico). Our models showed similar patterns with those spotted fever group rickettsiosis cases that derived from CDC in west and southwest states (Figure 6).

#### 4.1.5. *Amblyomma maculatum* (Gulf Coast Tick)

*Amblyomma maculatum* can cause spotted fever by transmitting the causative agent, *Rickettsia parkeri* [100], and tularemia by transmitting the bacterium *Francisella tularensis* [50]. This species is found in coastal areas of the southern United States, as well as regions bordering the Gulf of Mexico and the Caribbean [101]. The geographic distribution of *A. maculatum* extends west to southwestern Tennessee, Kansas, and Oklahoma, potentially thanks to cattle importations from established range areas [102]. Birds may also play a crucial rule in dispersing this species by carrying immature ticks into new areas during migration; such areas may include South and North Carolina, Virginia, Delaware, and eastern Maryland [103].

Our model projections showed suitable areas to include the species’ known range, and rather overly broadly northward to Missouri, Kentucky, southern Illinois, Ohio, and Indiana (Figure 2E). Under future conditions, models suggested that this species will see a range that is mostly stable in its presently suitable areas, with restricted areas of anticipated range reduction in Texas. Range expansions were anticipated by model transfers that would suggest the potential for range expansion along the entire northern edge of the species’ distribution, but the significance of these results is unclear given the general overprediction in the models for this species (Figure 2E).

**Figure 7:**
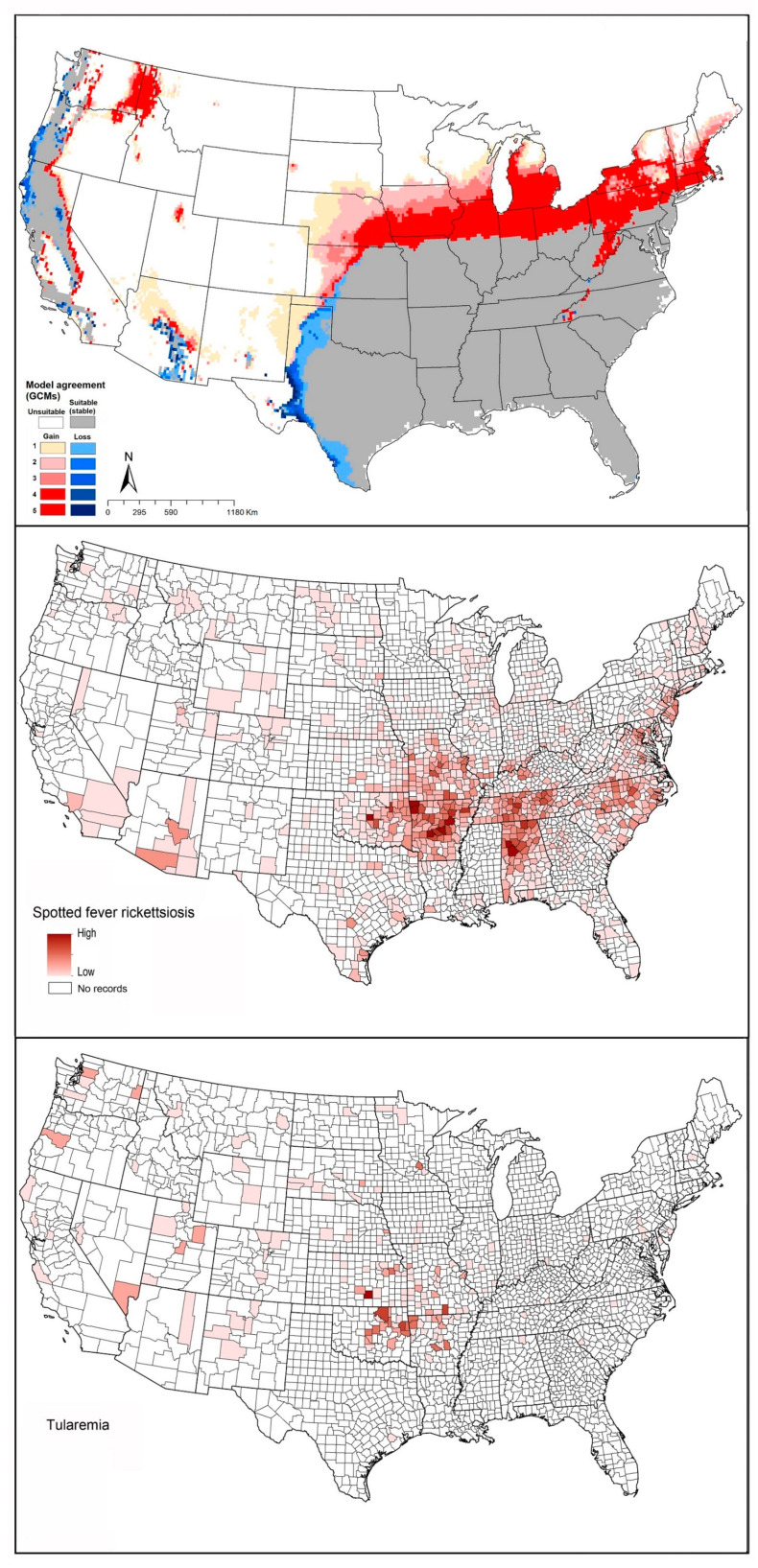
Geographic distribution of *Amblyomma maculatum* and the diseases that it likely transmits in the United States. Dark pink represents high incidence, light pink indicates low incidence. White represents no records.

Known cases of spotted fever group rickettsiosis were concentrated in Arkansas, Alabama, Tennessee, Oklahoma, Missouri, and North Carolina, all in regions known to hold populations of *A. maculatum*. Our models predicted all of those areas as suitable for the tick (Figure 7). Tularemia cases were more narrowly distributed, with most cases in Oklahoma, Arkansas, Kansas, and Missouri (Figure 7), although in large part farther north than the distribution of this tick species.

#### 4.1.6. *Amblyomma americanum* (Lone Star Tick)

*Amblyomma americanum* is a vector of ehrlichiosis (*Ehrlichia chafeenisis* and *E. ewingii*), tularemia (*F. tularensis*), viral diseases (e.g., heart virus, and Bourbon virus), and protozoans [104]. This species is apparently also associated causally with the poorly understood red meat allergy [105]. This species is considered as the most aggressive and important disease vector tick in the United States in view of its high population densities and nonspecific feeding habits [104].

The geographic distribution of this species covers much of the eastern United States. The species was first documented as occurring in New York in 1969, with small established populations on Long Island; after two decades, however, the species’ distribution had expanded rapidly to cover 46 of 62 New York’s counties [106]. By the end of the 1990s, its range had expanded to cover much of the northeastern United States [107], and in the Midwest to include Missouri, Nebraska, and Oklahoma [87,108,109]. Springer, Eisen [110] demonstrated that *A. americanum* is established in 653 counties across the southeastern and southcentral states, to include 32 states; 647 counties in 36 states reported (not necessarily established) the presence of this species.

Raghavan, Peterson (12) estimated the geographic distribution of *A. americanum* using ecological niche modeling in the context of current and future climate data. The current distribution of the species ranges from the east coast west to Kansas, Missouri, Oklahoma, Texas, southern Iowa, and Illinois (Figure 2F). Transferring models to future conditions, range expansions are anticipated in the northern United States, including in Iowa, Wisconsin, Michigan, and New England; southeastern and southern states are anticipated to see reductions in suitability, particularly in Florida, Alabama, Mississippi, Louisiana, western Texas, western Oklahoma, and Kansas (Figure 2F).

**Figure 8:**
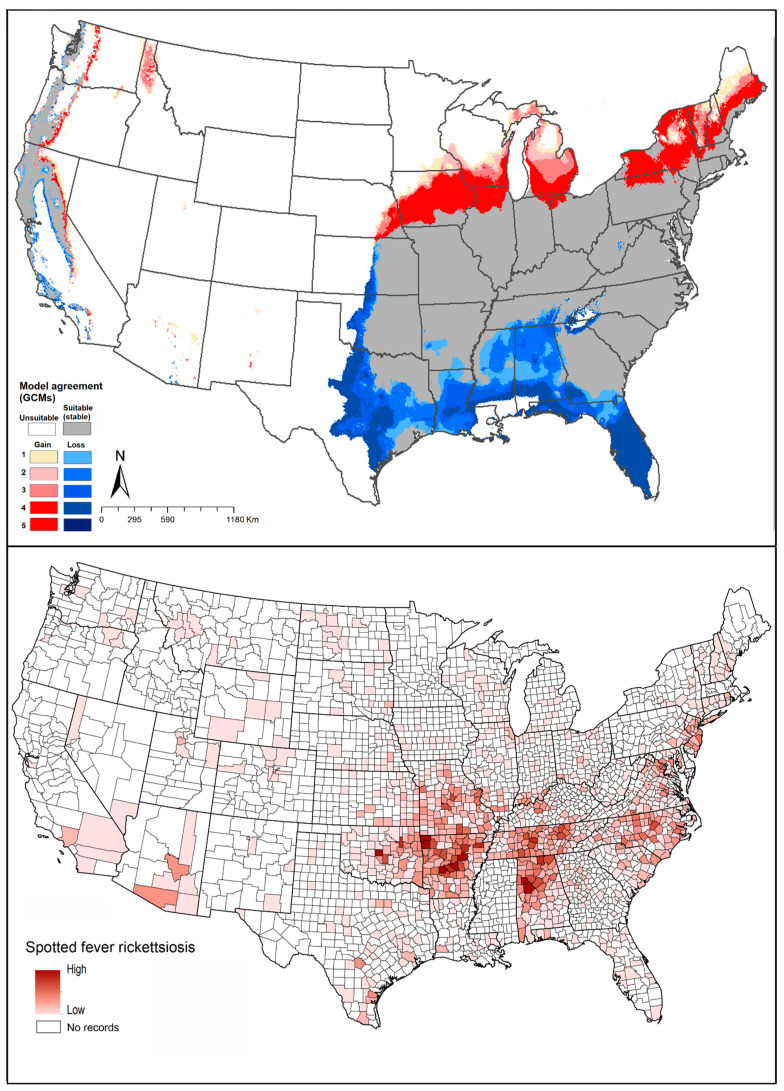
Geographic distribution of *Amblyomma americanum* and the disease that it likely transmits in the United States. Dark pink represents high incidence, light pink indicates low incidence. White represents no records.

The geographic distribution of known tularemia cases was closely similar to the geographic distribution of *A. americanum* (Figure 8). However, this pathogen species also overlapped broadly with *A. maculatum* (Figure 2E).

#### 4.1.7. *Dermacentor andersoni* (Rocky Mountain Wood Tick)

*Dermacentor andersoni* is a known vector of Rocky Mountain spotted fever (caused by *Rickettsia rickettsii*) [111], bovine anaplasmosis (caused by *Anaplasma marginale*) [112], Colorado tick fever (caused by *Coltivirus*) [113], and tularemia (caused by *Francisella tularensis*) [114]. This species can be found in the western United States and Canada at elevations of 1200–3000 m or higher [115].

Based on an approximate map provided by the CDC, this species is found in Washington, Oregon, California, Idaho, Montana, Wyoming, Nevada, Utah, and Colorado, as well as more restricted areas of North Dakota, South Dakota, and Nebraska [116]. Our models showed that suitable areas for this species extend from western and Midwestern states, and central and western Canada, where the species is known to have populations. Modeled, climate-based predictions extended into the eastern United States as well, however, although no records of this species are available from those areas (Figure 2G). Modeled transfers to future conditions suggest potential for range expansion in more northern parts of Canada, and into more restricted areas in the eastern United States (Figure 2G). Potential for reduction in suitable areas was mostly in the Midwest, including Kansas, Missouri, Nebraska, and South Dakota; in the Southeast, including Arkansas, Tennessee, Kentucky, Louisiana, Mississippi, and Alabama; and restricted areas in the west, including parts of New Mexico, Arizona, Utah, Nevada, California, Oregon, and Washington (Figure 2G).

**Figure 9:**
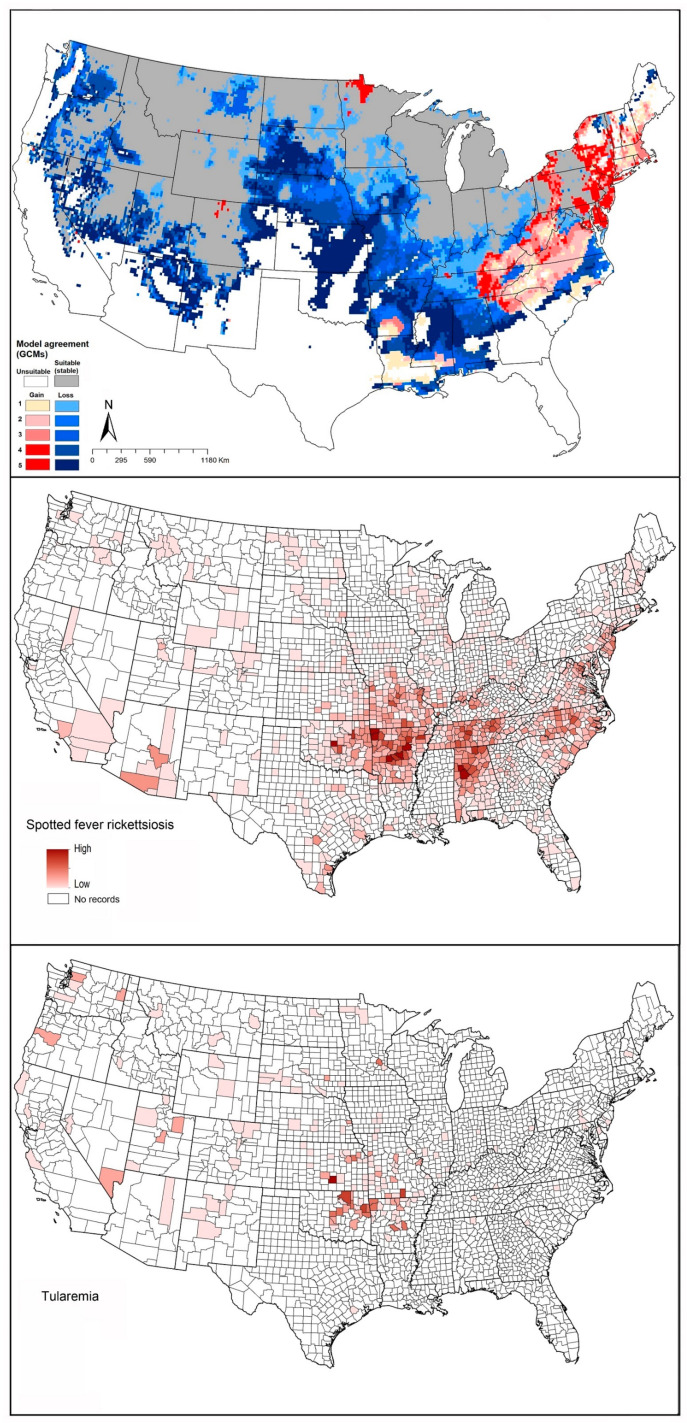
Geographic distribution of *Dermacentor andersoni* and the diseases that it likely transmits in the United States. Dark pink represents high incidence, light pink indicates low incidence. White represents no records.

Reported cases of spotted fever rickettsiosis showed patterns coincident with the southern portion of the modeled potential geographic distribution of *D. andersoni* (Figure 9). Similarly, tularemia cases showed general coincidence with the southern portion of the suitable areas of this species (Figure 9).

#### 4.1.8. *Dermacentor variabilis* (American Dog Tick)

*Dermacentor variabilis* is the vector of *Rickettsia rickettsii*, which causes Rocky Mountain spotted fever; it can also transmit *Francisella tularensis* and *Coxiella burnetii* [117]. Dermacentor variabilis uses particular species of small mammals as hosts for larval and nymph stages [2]; however, this species uses numerous hosts in the adult stage, including dogs, white-footed mice (*Peromyscus leucopus*), deer mice (*P. maniculatus*), and meadow voles (*Microtus pennsylvanicus*). This species does not have the ability to survive at mean temperature below 0 °C during December to February [118]. James and colleagues found that both elevation and temperature impact the presence of *D. variabilis* [119].

Boorgula et al. (2020) models showed suitable areas for this species including almost all of the United States except parts of northwestern and southwestern states. Model-predicted expansion areas are along the northern range limit, including some northwestern states in the United States and across Canada. Wood et al. (2016) reported that the geographic distribution of this species has expanded northward into Canada, particularly in Saskatchewan and Manitoba. Reductions in suitability were anticipated in model outputs in restricted areas across the Midwestern United States (Figure 2H).

**Figure 10:**
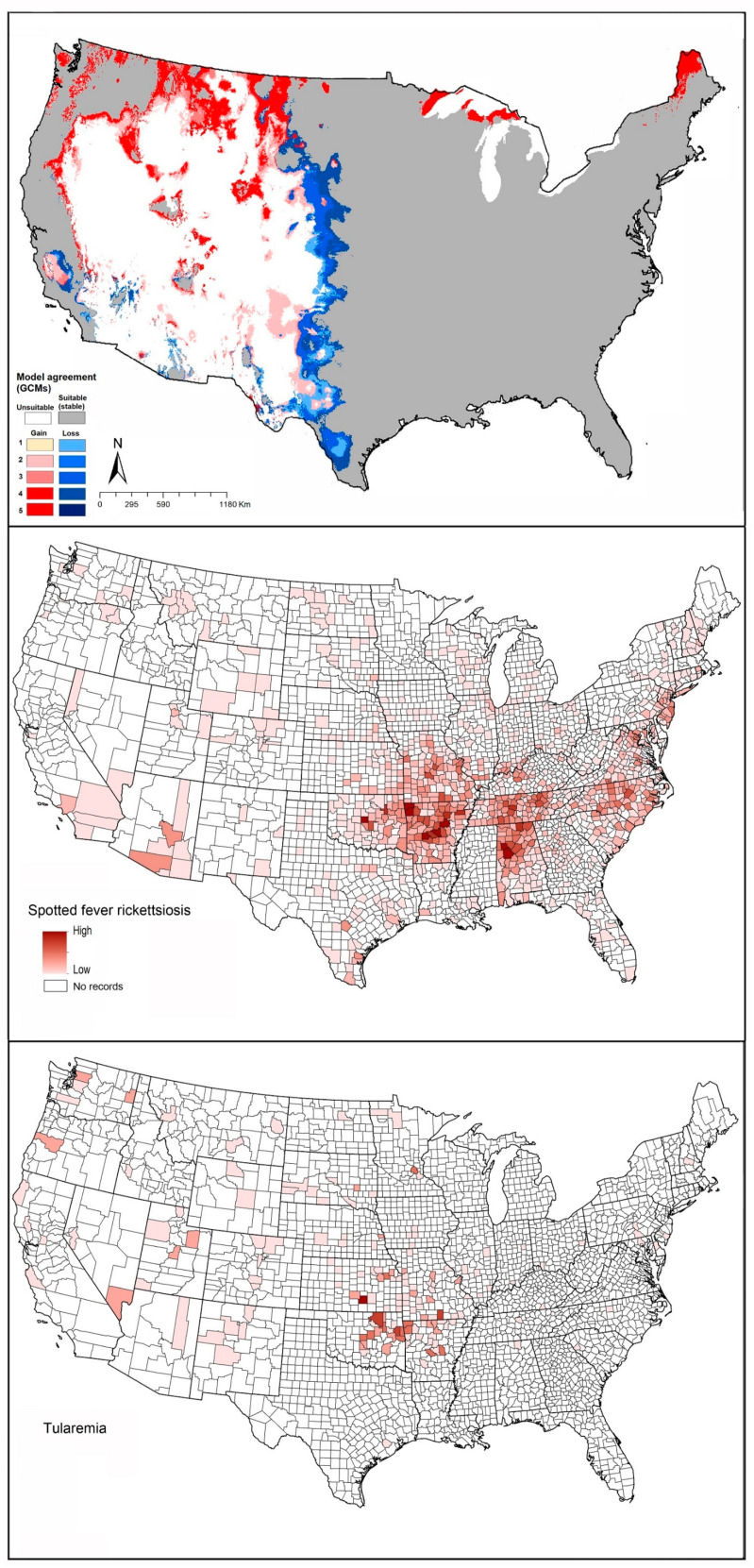
Geographic distribution of *Dermacentor variabilis* and the diseases that it likely transmits in the United States. Dark pink represents high incidence, light pink indicates low incidence. White represents no records.

The potential geographic distribution of this species coincided reasonably closely with the known distribution of cases of spotted fever rickettsiosis and tularemia (Figure 10).

## 5. Discussion

This contribution is designed to provide a broad overview of climate change implications for tick-associated disease risk across the United States and adjoining regions of southern Canada. Our work included reviewing and revisiting results of three published papers from our group, on *A. americanum* [12], *Rhipicephalus sanguineus* [14], and *D. variabilis* [13]. For a further five species, we present results herein from our modeling efforts. In all cases, our models took advantage of all occurrence data for the species that we found to be available as digital accessible knowledge [120,121]. We followed the most up-to-date methods in ecological niche modeling to assess potential geographic distributions, using detailed model-selection approaches implemented in the kuenm R package [64]. Our model transfers all included multiple GCMs and RCPs, to allow detailed consideration of model uncertainty, as well as analysis of extrapolative conditions via the MOP metric [122].

**Figure 11:**
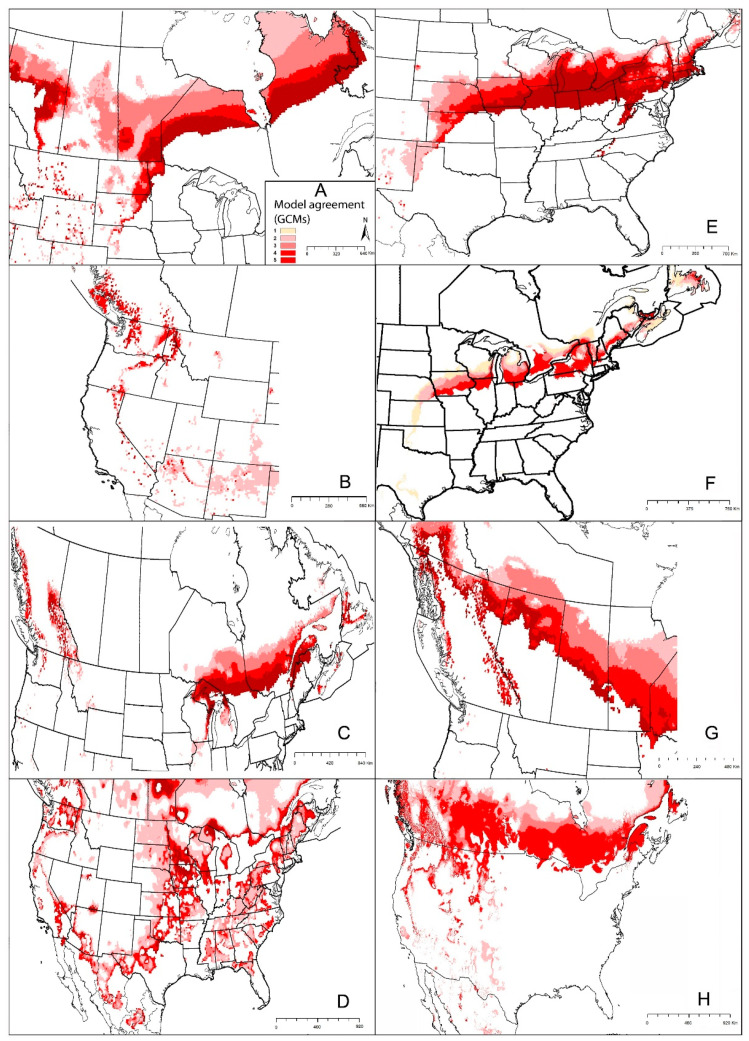
Summary of the potential expansion in geographic distribution of eight medical important ticks using five GCMs (CCCMA-CANESM2, CESM1-BGC, GISS-E2-R, IPSL-CM5A-MR, CCSM4) (under RCP4.5). Dark red indicates high agreement among models GCMs. (**A**) *Ixodes scapularis*, (**B**) *I. pacificus*, (**C**) *I. cookei*, (**D**) *Rhipicephalus sanguineus*, (**E**) *Amblyomma maculatum*, (**F**) *A. americanum*, (**G**) *Dermacentor andersoni*, (**H**) *D. variabilis*.

The proposition that continued temperature rise would expand suitable ranges for many species northward is a useful general guide. Here, we considered only climatic factors (temperature and precipitation) as crucial factors that may affect geographic distributions of tick species [2], and may influence ticks’ development, activity, and behavior [123,124,125,126]. Diyes et al. [125] for example showed that oviposition period for female *D. variabilis* can be 10–21 days at 25 °C and 95% relative humidity, and that larvae can survive under temperatures of 32 °C for 100 days but only 25 days at 5 °C. However, other factors will certainly also modify, enable, or slow range shifts of ticks and tick-associated disease risk such as availability and abundance of hosts [17], human-mediated movements of animals from established distributional areas [102], human-mediated land-use change, and human outdoor activities that may or may not lead them to interact with tick habitats [127]. Our models showed that most of the tick species will show a dominant pattern of range stability (Figure 2), but with a tendency to advance northward (Figure 11). In a few species, we also noted a tendency to retract from southern portions of the range or from interior sectors of the species’ ranges; a few species (e.g., *I. cookei*, and *A. americanum*) are expected to be more affected by temperature increases in terms of range retractions along the southern limits of their ranges (Figure 1 and Figure 2, Figures S1 and S2).

The eastern United States appears to hold more medically important tick species, which may make it present higher risk of tick-associated disease transmission than in the central and western United States. For instance, in this study, four tick species had closely similar geographic distributions in the eastern United States: *I. scapularis*, *A. americanum*, *A. maculatum*, and *D. variabilis*; *I. cookei* coincides partly with the other four species in the northeastern United States (Figure 2 and Figure S1). These overlaps can cause difficulties to public health in terms of controlling different tick populations and discovering the source of the pathogens.

One of the most tick-associated disease that caught our attention as an exception to the basic patterns was babesiosis. For this disease, the case-occurrence data showed a geographic distribution that covered broad areas, including areas that are not known to hold either of the vector ticks (*I. scapularis* or *I. pacificus*), especially in Montana, Wyoming, Colorado, and New Mexico [73] (Figure 2, Figure 3 and Figure 4). Those cases may therefore represent transmission by other tick species, such as *I. dammini*, or by contaminated blood transfusion [128]. Similarly, we noted POWV cases documented from areas that are apparently not suitable for the main vector tick *I. cookei* (Figure 5). These discords between human case distributions and vector species’ geographic distributions echo previous work with poorly known sandfly species that are vectors of leishmaniasis in Mexico [129].

Recent years have seen increased awareness of the dangerous potential impacts of various tick-borne pathogens transmitted by the eight hard tick species analyzed herein (and probably several others) on human health. Clearly, awareness and prior knowledge by medical personnel lend considerable impetus to diagnosis and documentation of cases of those diseases. For example, increasing numbers of cases of babesiosis in Maine and New Hampshire (from 909 in 2012 to 2074 in 2015; [130]) may reflect increased awareness in the public health system as regards this disease, or it may reflect real geographic or population expansion of the tick vector. These questions also underline the importance of case documentation of such diseases including the place where the patient is exposed, any travel history, and blood transfusion history, which provide crucial details on the geographic provenance of the infection [131].

Although ecological niche modeling is considered a powerful tool in understanding the geography of pathogens, vectors, and hosts, some caveats and limitations should be considered. Specifically, complications in their use include (1) nonequilibrium distributions, such that a species does not occupy the full extent of its climatically suitable area owing to dispersal limitations or biotic interactions; the effect of this situation is that models can be mis-tuned, and often will be overly restricted in their predictions of distributional potential. Additionally, (2) biases in sampling among the input occurrence data, such that the data are concentrated in some regions more than other regions, can bias model results. Finally, (3) occurrence data can show considerable variation in their spatial resolution, which again can cause difficulties in model outputs. These complications indeed can compromise ecological niche model outputs [78,132], but have been mitigated to every extent possible in the methodologies employed in developing the models discussed in this contribution.

## Figures and Tables

**Table 1:** Summary of the seasonal activity, geographic distribution, and habitat of the eight tick species.

Tick Species	Primary Active Season	Geographic Distribution	Primary Habitat	Source
*Ixodes* *scapularis*	spring, summer, and fall	widely distributed across eastern United States	wooded vegetation, and sandy soils	[8,80]
*I. pacificus*	spring, and summer	Pacific coast of North America.	gambel oak, juniper (*Juniperus* spp.), sagebrush, and mixed grass habitat	[81,82]
*I. cookei*	summer	northeastern United States, southeastern Canada.	host’s nest or burrow	[83,84]
*Rhipicephalus sanguineus*	all seasons	broad, worldwide in the tropics and subtropics	diverse habitats, can live indoor	[14,85,86]
*Amblyomma maculatum*	spring and summer	Caribbean, south and central United States, Mexico, West Indies, Colombia, Venezuela, Peru	grasslands	[87,88]
*A. americanum*	summer and fall	Coastal areas along the Atlantic Ocean and Gulf of Mexico.	oak and pine forests	[8,87,89,90]
*Dermacentor andersoni*	spring	Southwestern Canada; Rocky Mountain states in the United States, at high elevations (1300–3000 m)	wooded habitat, grassland, low-growing vegetation	[8,91,92]
*D. variabilis*	spring and summer	Eastern United States, Pacific coast	mixed upland and mixed-oak, hickory-dominant forest	[93]

## Data Availability

Under personal communication and data agreement, we obtained detailed data (i.e., county-level totals of case numbers) for five tick-associated diseases for 2017—these data were kindly provided by Kristen Nichols Heitman, and Amy Schwartz (Centers for Disease Control and Prevention).

## Supplementary Materials

The following are available online at https://www.mdpi.com/2075-4450/12/3/225/s1, File S1. Full details on the methods. Figure S1. Summary of the potential geographic distribution of eight medical important ticks (under RCP4.5). Gray represents stable suitable areas. Red indicates expansion suitable areas under future conditions (Dark red = high model agreement, light red = low model agreement). Blue and green indicates suitable in current time, but not suitable in future (Dark blue = high model agreement, light blue = low model agreement). Figure S2. Summary of the potential reduction in geographic distribution of eight medical important ticks (under RCP4.5). Dark blue indicates high agreement among models GCMs. (**A**) *Ixodes scapularis*, (**B**) *Ixodes pacificus*, (**C**) *Ixodes cookei*, (**D**) *Rhipicephalus sanguineus*, (**E**) *Amblyomma maculatum*, (**F**) *Amblyomma americanum*, (**G**) *Dermacentor andersoni*, (**H**) *Dermacentor variabilis*.
